# Empowering Women Through Knowledge: A Systematic Review of Literature on Menstrual and Reproductive Health Literacy

**DOI:** 10.1177/24731242251363080

**Published:** 2025-08-14

**Authors:** Ashleigh Hansen, Jessica Bayes, Janet Schloss

**Affiliations:** Faculty of Health, National Centre for Naturopathic Medicine, Southern Cross University, Lismore, New South Wales, Australia.

**Keywords:** adult women, menstrual health, menstruation, reproductive health literacy

## Abstract

**Background:**

Reproductive health, including menstrual health, is a critical element of the overall well-being of women. Knowledge of menstrual health increases personal empowerment and self-care. This review explores adult women’s knowledge of menstrual and reproductive health.

**Methods:**

A systematic literature review was conducted across ProQuest, PubMed, MEDLINE, Scopus, CINAHL, and AMED, targeting original, peer-reviewed articles published between 2013 and 2023. Following the Preferred Reporting Items for Systematic Reviews and Meta-Analysis Protocols 2020 guidelines, 649 articles were screened. Eighteen cross-sectional studies met the inclusion criteria after critical appraisal using the Joanna Briggs Institute checklist for analytical cross-sectional studies.

**Results:**

Mean percentages of overall correct knowledge were provided in a limited number of studies, with scores ranging between 35.6% and 57.3%. In this review, mean values were calculated to represent categorical analysis of adult women’s correct knowledge of ovulation, menstruation, and female physiology. The following values represent women’s correct knowledge of these factors: timing of ovulation (43.7%), definition of ovulation (75.3%), identify physical signs of ovulation (52.4%), definition of menstruation (92.8%), identify menstrual cycle length (58.9%), identify duration of menstruation (44.6%), identify physical changes that might occur 2 weeks prior to menstruation (76.8%), identify female reproductive anatomy (68.9%), identify the term reproductive “hormones” (37%); identify the hormone estrogen (30.4%), identify the hormone progesterone (24%), correct knowledge of reproductive functional biology (51.2%), and correct knowledge of factors affecting reproductive health (62.8%).

**Conclusion:**

Adult women’s knowledge of reproductive and menstrual health remains lower than expected due to various personal, cultural, and social factors. Developing educational and health promotion interventions is crucial to improving women’s reproductive knowledge globally.

## Introduction

Menstrual health refers to well-being related to the menstrual cycle, and from a health literacy perspective, it involves the ability to obtain, understand, and act on health care information to make informed health decisions, defining menstrual health literacy as knowledge acquisition and application specific to menstrual health. An individual’s level of health literacy is one of the most crucial and vital factors in determining their own health perception and their health service use.^1^ Among other factors, adequate knowledge and awareness of basic elements of female reproductive health is crucial for women to be active agents of their own bodily autonomy, health perception, and self-agency in health care access.^2^ The female reproductive system is comprised of interconnected organs, including ovaries, fallopian tubes, a uterus, vagina, and vulva, as well as a complex hormonal system comprising a multitude of hormones, most importantly, estrogen and progesterone.^3^ The functional activity of the reproductive system encompasses the menstrual cycle, first occurring during puberty at menarche and continuing until menopause, as well as pregnancy and childbirth.^3^ Good menstrual health is crucial to women’s overall well-being, influencing biopsychosocial factors such as the impact of work stress and dysmenorrhea, or culturally appropriate social inclusion during menstruation, creating a unique female experience of health.^4–6^

The World Health Organization defines reproductive health as a state of complete physical, mental, and social well-being, and not purely the absence of disease or dysfunction, in all aspects of the reproductive system, its functions, and processes, as they exist in all phases of human life.^7^ Access to sexual and reproductive health care is also a fundamental human right.^8^ In reality, the reproductive health of women is subject to the quality and availability of health care, the cultural and socioeconomic development of the region in which they reside, and, critically, the position women hold in that society to live and thrive.^9^ Documented barriers for U.S. women to access reproductive health care include financial cost or lack of insurance, difficulty securing an appointment or attending a clinic, lack of a regular physician, and fear of poor confidentiality of services, with recent evidence suggesting an increase in the number of barriers between 2017 and 2021.^10^ Migrant women and women with disabilities, particularly those in low-income countries, are exposed to similar financial obstacles, yet further disparities exist in language barriers, lack of information, and discrimination when seeking access to reproductive health care.^11,12^

Arguably, the most defining factor for evaluating women’s access and experience of reproductive health care is their existing level of menstrual and reproductive health literacy.^1,2^ Recent investigation of adolescent populations of women has shown a deficit in knowledge pertaining to menstrual and reproductive health, though the evidence offers valuable data for further research and development of interventions to promote education and empowerment.^13^ School-based interventions have had particular focus for the improvement of reproductive health literacy to promising results.^14,15^ While women’s health literacy has undergone systematic review, and reproductive knowledge of adolescent female populations has garnered some research attention, a review of literature concerning adult women’s menstrual and reproductive health knowledge has yet to be conducted.^16^ This systematic literature review explores adult women’s knowledge of menstrual and reproductive health, in particular assessing their awareness of anatomical, biological, or hormonal aspects of the reproductive system and menstrual cycle.

## Methods

A protocol was developed according to the Preferred Reporting Items for Systematic Reviews and Meta-Analysis Protocols (PRISMA-P) 2020 statement.^17^ The review was registered with the International Prospective Register of Systematic Reviews, PROSPERO (Registration ID: CRD42023462460).

### Inclusion/exclusion criteria

The review included English-language studies in which the population were adult women, who were surveyed for their knowledge of anatomical, biological, or hormonal aspects of the reproductive and/or menstrual cycle. Exclusions for this study were adolescent females and studies focused on other aspects of reproductive or menstrual awareness, including menstrual hygiene practices and/or social, political, or religious attitudes around menstruation.

### Search terms

The selection of appropriate search terms was determined through a process of testing and refinement of specificity and relativity to the subject matter. The most relevant terms used in database searching were “knowledge; literacy; health literacy; menstruation; menstrual cycle; menses; reproductive; fertility; fertility awareness.” The search filters applied were “2013–2023; English language; peer reviewed.”

An example of search terms, taken from PubMed, is as follows: ((((knowledge) OR (literacy)) OR (health literacy) AND (y_10[Filter])) AND (((menstruation [MeSH]) OR (menstrual cycle)) OR (menses) AND (y_10[Filter]))) AND (((reproductive[MeSH Terms]) OR (Fertility[MeSH])) OR (Fertility awareness) AND (y_10[Filter])). A full list of the search strings for each database can be found in the supplemental information (see Supplementary Table S1).

### Databases

The databases included in the literature search were ProQuest, PubMed, Medline, Scopus, CINAHL, and AMED. Database selection was determined by the quality of their health and human sciences literature portfolio.

### Main outcomes

This review aimed to critically evaluate the existing literature that explores reproductive health literacy, including women’s knowledge, comprehension, and consciousness regarding menstrual and reproductive health.

### Data extraction

Data extracted from full-text articles were presented in tables, including study details such as authors, publication year, study design, investigated outcomes, and measurement tools. Participant characteristics—age, sex, and nationality—were documented alongside their reported knowledge of the reproductive and menstrual cycle. Results data, including effect measures and *p*-values, were summarized along with study conclusions. Where applicable, subgroup analyses examined ovulation, menstruation, female reproductive anatomy and physiology, and broader factors affecting reproductive health. Data extraction was conducted by A.H., with thematic concepts determined in collaboration with all authors to ensure consistency and accuracy in analysis and reporting.

### Risk of bias

The Joanna Briggs Institute (JBI) critical appraisal tool assessed risk of bias and evaluated study quality in this cross-sectional review. A JBI score above 70% indicated high-quality research, 50–70% medium quality, and below 50% poor quality. Since this review focused on women’s knowledge rather than a specific condition, the fourth JBI checklist item was excluded, with percentage scores calculated from the remaining seven items. Risk of bias outcomes played a key role in determining whether studies met the inclusion criteria for this review.

## Results

The full data analytics and results are presented in Table 1.

**Table 1.** Data Extraction and Summary of Results

Author/year	Country	Design	Participants	Outcomes	Measurement tools	Results^a^	Conclusions
Akizuki (2023)^18^	Japan	Cross-sectional study	First-year undergraduate education students, female (152)	Fertility, including factors impacting fertility	1. Cardiff Fertility Knowledge Scale (CFKS)	1. The mean percentage of correct answers to the CFKS in this study was 52.9%. Factors impacting fertility: age decline (87.5%); referral time (infertile) (42.8%); smoking (88.8%); older age (65.8%); being healthy (61.2%); never having period (67.8%); overweight (23%).	The results suggest that participants’ knowledge of fertility is insufficient overall, varies according to the specific topic, and may not be based on scientific evidence.
				Basic reproductive knowledge	2. Three extra questions developed to assess basic female reproductive knowledge	2. Reproductive knowledge: timing of ovulation (27.6%); primordial follicle promotion (4%); timing of menopause (70.4%).	
Ameade (2016)^19^	Ghana	Cross-sectional study	Female undergraduate students studying health sciences (293)	Knowledge of menstruation	1. Ten questions developed to assess menstrual physiology, female anatomy, and menstrual hygiene	1. Overall menstruation knowledge (57.3%); definition menstruation (92.8%); normal menstrual cycle interval (75.8%); fertile window (78.5%); hormones responsible for menstruation (26.3%); source of menstrual blood (55.9%); menstruation duration (39.6%); pregnant during menstruation (29.4%); menopause age (57.7%), poor menstrual hygiene leads to infection (95.9%).	Female university students possessed average knowledge of menstruation but they practiced good menstrual hygiene.
				Menstrual hygiene practices	2. Menstrual hygiene practice assessed using six questions	2. Use of sanitary pad (100%); frequency of sanitary pad change (2–3 times; 89.4%); cleaning genitals after urinating while menstruating (69.3%); correct disposal of sanitary pad (93.2%); bathe with soap and water on first day of menses (94.5%); bathing increases during menstruation (34.5%).	
Ayoola (2016)^20^	United States	Cross-sectional study	Women 18 years and older, nonpregnant, from Medically Underserved Populations (125)	General knowledge of reproductive system and menstrual cycle	Knowledge of Female Body (KFB) scale	Composite KFB score ranged from 0 to 25 for this population with a mean score of 15.1 (SD = 5.09). Anatomical structures important for reproduction—ovaries (86.4%), uterus (82.4%), fallopian tubes (78.4%); pregnancy occurs when egg is fertilized by sperm (82.4%); menstrual flow 2–8 days (80.8%); possible physical changes in last 2 weeks of cycle—bloating (68.8%), cramping (84.8%); menstrual cycle hormones—progesterone (24%), estrogen (30.4%); length of menstrual cycle 20–36 days (50.4%); hormones prepare uterus for pregnancy (53.6%); number of eggs released at ovulation (20.8%); ovulation definition (52.8%); timing of ovulation (32.8%); lifespan of egg and sperm (37.6%).	Sixty-eight percent of the women had a low knowledge of female reproduction and its associated changes.
Chawlowska (2020)^21^	Poland	Cross-sectional study	Female university students, 18–29 years (456)	Reproductive health literacy, including fertility awareness	Self-developed survey of 20 questions	Average all questions (55.8%); knowledge of all fertility signs (7%): libido increase (42.3%), ovulation pain (44.3%), soft cervix (46.9%), clear stretchy mucus (59.4%). Identified signs NOT associated with fertile window: whitish sticky mucus (73.9%), hard cervix (91.2%), menstrual pain (97.4%); knowledge of all adverse factors on fertility (8.1%): long-lasting physical effort (37.5%), irregular circadian rhythms (60.3%), drastic diet changes (61.2%), smoking (91%), diseases (93.4%), stress (95.6%); definition menopause (20.8%); cycle temperature changes (40.4%); ovum lifespan (46.3%); frequency of ovulation (54.8%); female fertility lifespan (46.3%); timing of ovulation (59.2%); length of cycle (59.2%); irregular cycle impact fertility (75.7%); fertile window (73.7%); definition fertility (75.7%); first day of cycle (86%); definition ovulation (97.8%).	General knowledge of respondents would be rated as average.
Fowler (2023)^22^	United States	Cross-sectional study	Nonsterilized, English speaking women aged 18–29 years (1,779)	Knowledge of reproductive biology: (1) age-related fertility decline, (2) fertile period, (3) egg supply	Self-developed and tested survey of 76 questions, 19 on fertility knowledge, and 3 of those questions selected for investigation in this study	Overall knowledge: (1) age-related fertility decline (62.4%); (2) fertile period (59.2%); (3) ovaries and egg production (44.7%).	Young U.S. women have incomplete knowledge of aspects of their reproductive biology, especially those from economically or socially marginalized groups.
Getahun (2020)^23^	Ethiopia	Cross-sectional study	Women, aged 15–49 years (15,683)	Knowledge of ovulation period among reproductive women	2016 Ethiopian Demographic Health Survey	Timing of ovulation (23.6%).	Knowledge of ovulation period among reproductive women was low.
Halleran (2022)^24^	Canada and United States	Cross-sectional study	Women of reproductive age 18–45 years (102), struggling to conceive without medical intervention for ≥12 months	Knowledge about basic human fertility	Fertility Knowledge Questionnaire	Cervical secretions are fertile sign (80%); normal for menstrual cycle to be shorter than 28 days (90%); after having a baby, pregnancy can only happen when period returns (91%); ovulation signs—increase in body temperature, pain in abdomen near ovary, cervical secretions (69%).	Women struggling to conceive appear to have generally adequate fertility knowledge, possible misinformation about fertile window.
Hamdanieh (2021)^25^	Lebanon	Cross-sectional study	Single, unmarried women living in Lebanon, aged 17–55 years (491)	Menstruation and its abnormalities	Questionnaire; menstruation section consisting of 12 questions	Overall menstruation knowledge (35.6%; adequate knowledge calculated to be ≥39%); average duration of menstruation (24.9%); menstrual cycle duration (46.1%); ovulation timing (47.3%); causes of amenorrhea: pregnancy (81.8%), hormonal dysfunction (79%), stress and heavy exercise (53.7%), polycystic ovarian syndrome (59.1%), eating disorders (39.5%); causes of dysmenorrhea: stressful life events (47%), ovarian cysts and tumors (61.1%), can be accompanied by nausea, vomiting, fatigue (68.1%).	Inadequate knowledge concerning menstruation and its abnormalities.
Jean Simon (2023)^26^	Haiti	Cross-sectional study	Women of childbearing age in Haiti, aged 15–49 years (14,371)	Knowledge of ovulatory cycle	2016/2017 Haitian Demographic Health Survey, subsection “Women’s Questionnaire”	Overall knowledge of ovulatory cycle (24.1%).	Prevalence of correct knowledge of the ovulatory cycle is low among women of childbearing age in Haiti.
Lundsberg (2014)^27^	United States	Cross-sectional study	Women aged 18–40 years (1000)	Factors affecting fertility	Online survey (eight relevant questions)	Factors affecting fertility: painful periods (30.4%), alcohol (69.3%), STIs (69.4%), smoking (71.3%), irregular periods (72.7%), underweight (73.2%), obesity (74%), stress (90%).	Knowledge regarding ovulation and fertility is limited among this sample of reproductive-aged women in the United States.
				Knowledge of ovulation	Online survey (five relevant questions)	Knowledge of ovulation: normal menstrual cycle duration (76.2%), timing of ovulation (60.6%), fertile mucus (60.3%), basal body temperature rise after ovulation (74%), ovarian egg production (60%).	
Mahey (2018)^28^	India	Cross-sectional survey	Women seeking fertility treatment, aged 21–44 years (205)	Factors affecting fertility	Questionnaire (three relevant questions)	Factors affecting fertility: age >35 years (26%), highest risk factor for infertility >35 years (7.8%), contraceptive use (2.9%).	Significant, important gaps were identified in women’s knowledge and awareness regarding fertility practices.
				Knowledge of ovulation	Questionnaire (one relevant question)	Timing of ovulation (15.1%).	
Marsh (2014)^29^	United States	Cross-sectional study	African American women, 18–60 years (193)	Self-reported prevalence of heavy menstrual bleeding (HMB)	Questionnaire (one relevant question)	Women reporting menses as heavy or very heavy (41.8%).	The self-reported prevalence of HMB in the study population exceeded the national prevalence and was associated with a significant lack of HMB knowledge among study participants.
				Knowledge of menstruation	Questionnaire (three relevant questions)	Menstrual bleeding >7 days considered excessive (33.2%); normal menstrual cycle duration (14.9%); HMB has causative relationship with anemia (76.2%).	
Mengistie (2023)^30^	15 low-income African countries	Cross-sectional study	Women of reproductive age, 15–49 years (235,574)	Knowledge of the highest conception probability period during menstrual cycle	Demographic and Health Survey (one question assessing ovulation timing)	Ovulation occurs in the middle of the menstrual cycle (24.04%).	Knowledge of the highest conception probability period among women of reproductive age in low-income African countries was low.
Na Nakhon (2018)^31^	Thailand	Cross-sectional study	Women aged 18–45 years living in Bangkok metropolitan area (233)	Factors influencing fertility	Questionnaire-based survey	Age of start of female fecundity decline (15%); fertile window (23.6%); smoking (74.7%); alcohol consumption (79.8%); obesity (50.6%); sexually transmitted infections (77.3%).	Most reproductive age participants living in an urban area of Thailand incorrectly identified factors that influence fertility.
Patra (2018)	India	Cross-sectional study	Women aged 20–45 years who had ever experienced infertility and received treatment	Assess level of reproductive health knowledge	Reproductive Health Knowledge Index (RHKI)	The mean RHKI score (2.84, *p* < 0.001) was lower among women aged 36 years and above than in younger women. The highest mean RHKI score (9.39, *p* < 0.001) was in women with secondary education. Women with no education and those with uneducated husbands had low RHKI scores (1.22 and 1.39, respectively, *p* < 0.001). Women not exposed to media or never worked had low RHKI scores (2.09, *p* < 0.001; 3.83, *p* < 0.001). Those with poor Wealth Index or from certain communities also had low RHKI scores (2.11, *p* < 0.01; 4.2, *p* < 0.05).	
					Structured and semi-structured questionnaires	No results to this questionnaire were presented in the study.	
Sons (2023)^32^	United States	Cross-sectional study	Female undergraduate students aged 18–24 years (237)	Knowledge of female reproduction	KFB scale	Low knowledge score = 0–17, high knowledge score = 18–26. Mean KFB score 21.6 ± 2.0. The percentages of each specific question correctly answered was not recorded independently for female participants.	Undergraduate students have major reproductive knowledge gaps.
Sreepoorna (2020)^33^	United Arab Emirates	Cross-sectional study	Female Emirati students aged 18–25 years (493)	Reproductive health knowledge	Questionnaire	Very well/well level of reproductive health knowledge: periods (80.5%), uterus (56.6%), ovary (53.6%), hormones (47.8%), biology behind pregnancy (53.6%), biology behind childbirth (49%); Infertility knowledge: disease in ovaries is related to infertility (29.2%), absent/irregular periods are not normal (46.4%), abnormal hormones cause of infertility (44.4%), male hormones are present in women (38.6%).	This study provides insight into students’ low awareness of reproductive health and disorders such as PCOS.
				Awareness of PCOS	Questionnaire	Awareness of PCOS (38.4%).	
Szucs (2017)^34^	Hungary, Serbia, and Romania	Cross-sectional study	Female university students aged 18–28 years (2572)	Knowledge on the menstrual cycle	Questionnaire-based survey	The most correct answers were significantly higher among the students of health sciences than other courses: 86.0%, 71.5%, and 61.1% vs. 71.9%, 59.8%, and 43.2% in Serbia, Hungary, and Romania, respectively.	71.5% and 59.8% of the Hungarian, 86% and 71.8% of the Serbian, and 61.1% and 43.2% of the Romanian students of health sciences and students of other faculties had proper knowledge of the fertile period within a menstrual cycle.

^a^ Results indicate percentages of correct knowledge identified.

PCOS, polycystic ovary syndrome; SD, standard deviation.

### Identification of studies

**FIG. 1.**
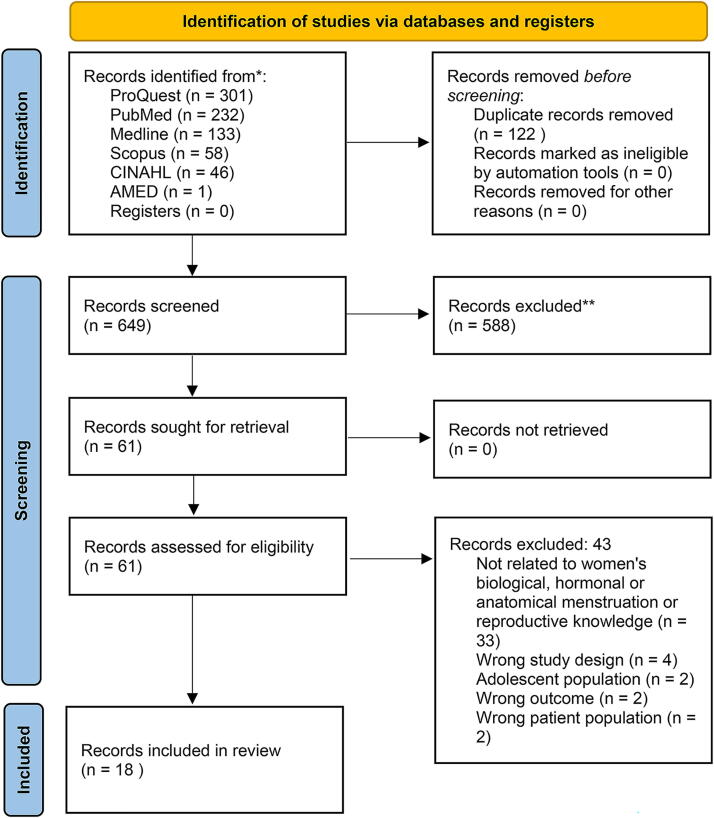
PRISMA diagram. PRISMA, Preferred Reporting Items for Systematic Reviews and Meta-Analyses.

The initial search identified 771 articles, which were entered into Covidence, a web-based platform for streamlining systematic reviews. After removing 122 duplicates, 649 articles were screened by title and abstract. Following full-text screening of 61 articles, 18 met the inclusion criteria and were included in this review. The primary reason for exclusion was insufficient data related to women’s knowledge of anatomical, biological, or hormonal aspects of menstrual and reproductive health. Two reviewers independently screened the articles, reaching consensus on eligibility at each stage in Covidence before progressing. The article search and review process is illustrated in Figure 1.

### Risk of bias results

The majority of articles described the study subjects and design in detail, with the exception of two.^28,29^ The area in which the majority of studies performed poorly was statistical analysis, specifically, in the identification and strategizing of confounding factors. Two studies (11%)^20,22^ identified confounding factors, and three studies (17%)^22,26,30^ discussed strategies for representation of confounding factors in the data. The most variable results in this assessment pertained to clearly defined inclusion criteria in the studies. Eleven of the studies (61%)^18,20–22,24,25,28,31–33,35^ clearly stated the inclusion criteria, four studies (22%)^19,23,27,34^ did not have clearly defined criteria, and the remaining three studies (16%)^26,29,30^ were unclear. Fifty percent of the studies accrued 5 points, the most common point score. Categorization of study quality according to the JBI tool is as follows: high quality (*n* = 11), medium quality (*n* = 6), and poor quality (*n* = 1). The majority of the studies were of high or medium quality; therefore, it was determined that the review would continue inclusive of all 18 articles. A brief summary is presented in Table 2, while the detailed critical appraisal of included studies using the JBI tool is provided in the Supplementary Data.

**Table 2.** Johana Briggs Institute Critical Appraisal for Cross-Sectional Studies Summary

Study ID (author/year)	Country	Sample size	Instrument	JBI quality score
Akizuki (2023)^18^	Japan	152	Cardiff Fertility Knowledge Scale (CFKS), plus three extra self-developed questions	High
Ameade (2016)^19^	Ghana	293	Self-developed questionnaire	Medium
Ayoola (2016)^20^	United States	125	Knowledge of Female Body (KFB) scale	High
Chawlowska (2020)^21^	Poland	456	Self-developed survey	High
Fowler (2023)^22^	United States	1779	Self-developed and tested survey	High
Getahun (2020)^23^	Ethiopia	15,683	2016 Ethiopian Demographic Health Survey	Medium
Halleran (2022)^24^	Canada and United States	102	Fertility Knowledge Questionnaire	High
Hamdanieh (2021)^25^	Lebanon	491	Self-developed questionnaire	High
Jean Simon (2023)^26^	Haiti	14,371	2016/2017 Haitian Demographic Health Survey, subsection “Women’s Questionnaire”	High
Lundsberg (2014)^27^	United States	1000	Self-developed online survey	Medium
Mahey (2018)^28^	India	205	Self-developed questionnaire	Medium
Marsh (2014)^29^	United States	193	Self-developed and tested questionnaire	Poor
Mengistie (2023)^30^	15 low-income African countries	235,574	Demographic and Health Survey	High
Na Nakhon (2018)^31^	Thailand	233	Self-developed and tested questionnaire from survey	High
Patra and Sayeed (2021)^35^	India	159	Reproductive Health Knowledge Index (RHKI)	Medium
Sons (2023)^32^	United States	237	KFB scale	High
Sreepoorna (2020)^33^	United Arab Emirates	493	Self-developed and tested questionnaire	High
Szues (2017)^34^	Hungary, Serbia, and Romania	2572	Self-developed questionnaire from survey	Medium

JBI, Joanna Briggs Institute.

### Overview/demographic data

The studies were conducted within the following regions: the United States and Canada (*n* = 5), Asia (*n* = 4), Africa (*n* = 3), Europe (*n* = 2), the Middle East (*n* = 2), and Central America (*n* = 1). The age range of participants across all studies was 15–60 years. Thirteen of the 18 studies (72%) calculated specific age range data, with the majority of those participants aged <30 years (80%), followed by 30–40 years (14%) and 40+ years (5%). Marital status was obtained in 50% of the studies, and on average, less than half (47%) of those participants were married.

There was a wide-ranging variability in the measurement tools adopted in each study. Of the 18 studies reviewed, 6 studies (33%) utilized a single preexisting validated measurement tool, 3 of those being National Demographic Health surveys.^20,23,24,26,30,32^ Two studies shared use of the same validated measurement tool, the Knowledge of Female Body (KFB) scale.^20,32^ Two studies (11%) combined existing measurement tools together with self-developed questionnaires.^18,35^ The remaining 10 studies (55%) developed unique questionnaires for their own investigative purposes, of which four produced tested and validated instruments.^22,29,31,33^

Seven studies determined overall knowledge scores; however, these results were calculated relative to the measurement tool adopted in the study. Mean percentages of correct knowledge were calculated in four of these studies, with scores ranging between 35.6% and 57.3%.^18,19,21,25^ One study adopted the Reproductive Health Knowledge Index with a scoring system between 0 and 11 (a score of 0–2 equaled no/low knowledge), with a calculated mean score of 2.84.^35^ The KFB scale, which ranges from 0 to 26 points, categorizes overall knowledge scores as follows: less than 70% is considered low knowledge (0–17 points), and greater than 70% is considered high knowledge (18–26 points).^20^ In the two studies that utilized this scale, the mean overall knowledge scores were 15.1 (indicating low knowledge)^20^ and 21.6 (indicating high knowledge).^32^

**FIG. 2.**
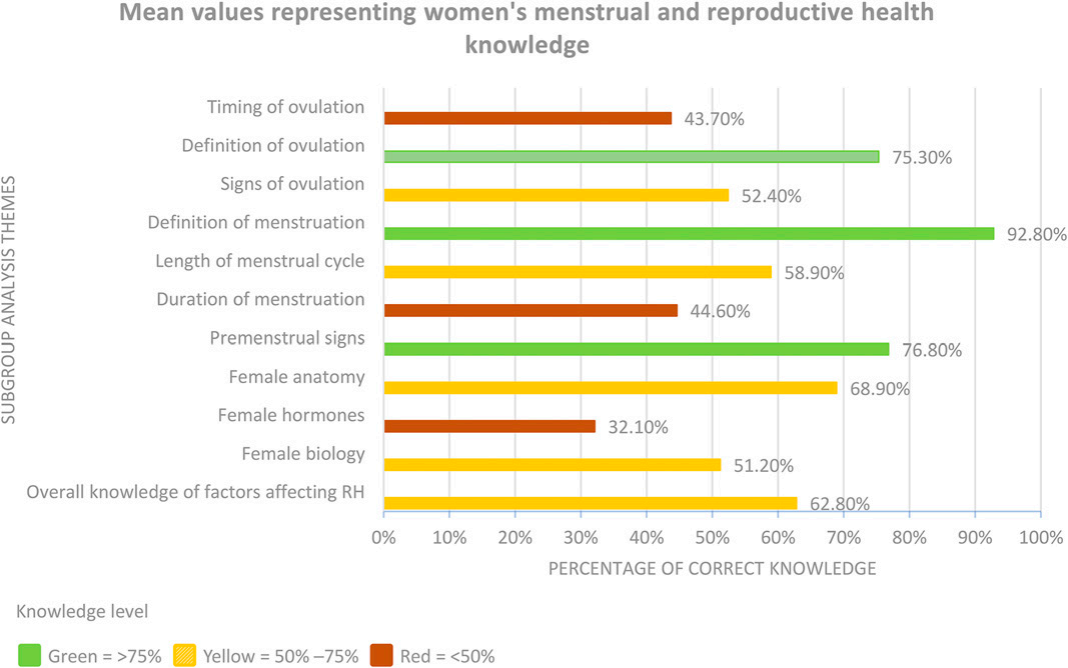
Overall mean values representing women’s correct knowledge across all subgroup analyses.

As the outcome measurement instruments used across the included studies varied considerably, direct comparison of data was not feasible. To address this, an analysis was undertaken via secondary means by identifying shared thematic content across the studies. Despite the variability, the outcome measures could be broadly grouped into four categories: (1) knowledge of ovulation, (2) knowledge of menstruation, (3) knowledge of female reproductive physiology, and (4) knowledge of factors affecting reproductive health. These categories were developed through a close reading and inductive synthesis of the survey instruments employed across the studies. By organizing the data into these overarching themes, it was possible to generate more meaningful comparisons and facilitate a coherent synthesis of findings. Figure 2 provides a visual summary of the research findings identified through subgroup analysis.

### Knowledge of ovulation

Ovulation, also referred to as the fertile window, was the primary outcome explored in 16 of the 18 studies (89%).^18–28,30–32,34,35^ Knowledge of the ovulatory window was assessed in three ways: timing of ovulation, definition of ovulation, and signs of ovulation (see Table 3). Knowledge of the timing of ovulation was the most common theme explored in the studies (83%), while the definition and signs of ovulation were each investigated in 17% of studies. The studies that questioned awareness of the timing of ovulation found that, on average, 43.7% of women had correct knowledge.^18–23,25–28,30–32,34,35^ On average, 75.3% of women questioned had correct knowledge of the definition of ovulation.^20,21,32^ Signs of ovulation, including cervical secretions, libido changes, and temperature changes, were explored in fewer articles, with a calculated average of 51.6% of women questioned demonstrating correct knowledge.^21,24,27^

**Table 3.** Results of Subgroup Analysis of Women’s Knowledge of the Timing, Definition, and Physical Signs of Ovulation/Fertile Window

Ovulation/fertile window			
Author/year	Timing of ovulation	Definition of ovulation	Signs of ovulation
Akizuki (2023)^18^	Timing of ovulation (27.6%)^a^		
Ameade (2016)^19^	Fertile window (78.5%)		
Ayoola (2016)^20^	Timing of ovulation (32.8%)	Definition of ovulation (52.8%)	
Chawlowska (2020)^21^	Timing of ovulation (59.2%); fertile window (73.7%)	Definition of ovulation (97.8%)	Knowledge of all fertility signs (7%): libido increase (42.3%), ovulation pain (44.3%), soft cervix (46.9%), clear stretchy mucus (59.4%); cycle temperature changes (40.4%)
Fowler (2023)^22^	Fertile window (59.2%)		
Getahun (2020)^23^	Timing of ovulation (23.6%)		
Halleran (2022)^24^			Cervical secretions are fertile sign (80%); ovulation signs—increase in body temperature, pain in abdomen near ovary, cervical secretions (69%)
Hamdanieh (2021)^25^	Timing of ovulation (47.3%)		
Jean Simon (2023)^26^	Fertile window (24.1%)		
Lundsberg (2014)^27^	Timing of ovulation (60.6%)		Fertile mucus (60.3%), basal body temperature rises after ovulation (74%)
Mahey (2018)^28^	Timing of ovulation (15.1%)		
Mengistie (2023)^30^	Timing of ovulation (24.04%)		
Na Nakhon (2018)^31^	Fertile window (23.6%)		
Patra (2018)	Fertile window (nil percentage)		
Sons (2023)^32^	Timing of ovulation (nil percentage)	Definition of ovulation (nil percentage)	
Szucs (2017)^34^	Fertile window (63%)		
Calculated mean percentage of correct knowledge	43.7%	75.3%	52.4%

^a^ Percentages indicate participants with correct knowledge.

### Knowledge of menstruation

Knowledge of menstruation was investigated in 8 of the 18 studies reviewed (44%).^19,20,24,25,27,29,32,35^ Five themes were presented across the literature: the definition of menstruation, length of menstrual cycle, duration of menstruation, premenstrual signs, and dysfunction of menstruation (see Table 4). The definition of menstruation was correctly identified by 93% of women surveyed.^19^ On average, 58.9% of women had correct knowledge of the length of the menstrual cycle.^19,20,24,25,27,29,32,35^ Duration of menstruation was correctly identified, on average, in 44.6% of the women surveyed.^19,20,25,29,32^ Women correctly identified symptoms of bloating (68.8%) and cramping (84.8%) as possible physical changes in the last 2 weeks of the menstrual cycle.^20^

**Table 4.** Results of Subgroup Analysis of Women’s Knowledge of the Definition, Length, and Duration of Menstruation Plus Premenstrual Signs and Dysfunction of Menstruation

Menstruation					
Author/year	Definition of menstruation	Length of menstrual cycle	Duration of menstruation	Premenstrual signs	Dysfunction of menstruation
Ameade (2016)^19^	Definition of menstruation (92.8%)^a^	Normal menstrual cycle interval (75.8%)	Menstruation duration (39.6%)		
Ayoola (2016)^20^		Normal length of menstrual cycle 20–36 days (50.4%)	Menstruation duration 2–8 days (80.8%)	Possible physical changes in last 2 weeks of menstrual cycle—bloating (68.8%), cramping (84.8%)	
Halleran (2022)^24^		Normal for menstrual cycle to be shorter or longer than 28 days (90%)			
Hamdanieh (2021)^25^		Normal length of menstrual cycle (46.1%)	Menstruation duration (24.9%)		Causative factors amenorrhea: pregnancy (81.8%), hormonal dysfunction (79%), stress and heavy exercise (53.7%), polycystic ovarian syndrome (59.1%), and eating disorders (39.5%). Causative factors dysmenorrhea: stressful life events (47%), ovarian cysts and tumors (61.1%), and that the condition can be accompanied by nausea, vomiting, fatigue (68.1%)
Lundsberg (2014)^27^		Normal length of menstrual cycle (76.2%)			
Marsh (2014)^29^		Normal length of menstrual cycle (14.9%)	Menstrual bleeding >7 days considered excessive (33.2%)		Women reporting menses as heavy or very heavy (41.8%); heavy menstrual bleeding has causative relationship with anemia (76.2%)
Patra (2018)		Normal length of the menstrual cycle (nil percentage)			
Sons (2023)^32^		Menstrual cycle length from the beginning of one period to the beginning of the next period is between 20 and 36 days (nil percentage)	Menstrual duration 2–8 days (nil percentage)	Possible physical changes during the last 2 weeks of menstrual cycle: bloating/cramping (nil percentage)	
Calculated mean percentage of correct knowledge	92.8%	58.9%	44.6%	76.8%	60.7%

^a^ Percentages indicate participants with correct knowledge.

A noteworthy secondary outcome measured was the investigation of women’s knowledge surrounding abnormal presentations of menstruation. In the case of dysfunction of menstruation, women who experience heavy menstrual bleeding had poorer knowledge of the normal duration of menstruation. A high proportion of the women surveyed (42%) reported menses as heavy or very heavy, but only 33% of those women correctly identified that menstrual bleeding longer than 7 days is considered excessive. A greater percentage of participants (76%) could correctly identify that heavy menstrual bleeding has a causative relationship with anemia.^29^

In contrast, women’s knowledge of causative factors associated with amenorrhea (absent menstruation) and dysmenorrhea (painful menstruation) was higher than anticipated. The results showed percentages of participants correctly identified amenorrhea to be related to the following factors: pregnancy (81.8%), hormonal dysfunction (79%), stress and heavy exercise (53.7%), polycystic ovarian syndrome (59.1%), and eating disorders (39.5%). Similarly, the participants’ knowledge of causes of dysmenorrhea: stressful life events (47%), ovarian cysts and tumors (61.1%), and that the condition can be accompanied by nausea, vomiting, and fatigue (68.1%).^25^

### Knowledge of female reproductive physiology

Eight of the 18 studies reviewed (44%) explored knowledge of female reproductive physiology.^19–22,27,32,33,35^ This topic was explored in three ways: knowledge of anatomical structures of the reproductive system, hormones of the reproductive system, and functional biology of the reproductive system (see Table 5). Knowledge of female anatomy was investigated in six studies (62.5%), with women’s correct identification of reproductive organs as average percentages: the ovaries (70%), fallopian tubes (78.4%), and uterus (69.5%), as well as correctly identifying the source of menstrual blood (55.9%).^19,20,32,33,35^

**Table 5.** Results of Subgroup Analysis of Women’s Knowledge of Reproductive Physiology Including Anatomy, Hormones, and Functional Biology of the Female Reproductive System

Female reproductive physiology			
Author/year	Anatomical structures of reproductive system	Hormones of reproductive system	Functional biology of reproductive system
Ameade (2016)^19^	Source of menstrual blood (55.9%)^a^	Hormones responsible for menstruation (26.3%)	
Ayoola (2016)^20^	Anatomical structures important for reproduction—ovaries (86.4%), uterus (82.4%), fallopian tubes (78.4%)	Menstrual cycle hormones—progesterone (24%), estrogen (30.4%)	Number of eggs released at ovulation (20.8%)
Chawlowska (2020)^21^			Ovum lifespan (46.3%); frequency of ovulation (54.8%)
Fowler (2023)^22^			Ovaries egg production across lifespan (44.7%)
Lundsberg (2014)^27^			Ovarian egg production across lifespan (60%)
Patra (2018)	Knowledge of the male and female reproductive organs; knowledge of exact place in a woman’s body where a baby grows (nil percentage)		
Sons (2023)^32^	Structures in a woman’s body important for reproduction—Ovary, Fallopian tubes, Uterus (nil percentage)	Hormones important in the menstrual cycle: progesterone/estrogen (nil percentage)	Ovum lifespan (nil percentage)
Sreepoorna (2020)^33^	Uterus (56.6%), ovary (53.6%)	Hormones (47.8%)	Awareness of term: Periods (80.5%), biology behind pregnancy (53.6%), biology behind childbirth (49%)
Calculated mean percentage of correct knowledge	Overall knowledge (68.9%) Ovaries (70%) Uterus (69.5%) Fallopian tubes (78.4%)	Overall knowledge (32.1%) “Hormones” (37%)	Overall knowledge (51.2%)

^a^ Percentages indicate participants with correct knowledge.

There were clear inconsistencies as to the method of investigating women’s knowledge of hormones of the reproductive system when comparing studies. One study sought women’s awareness of the term “hormones,” of which 47.8% responded correctly.^33^ Another study questioned whether women were aware that hormones were responsible for menstruation, in which 26.3% of women surveyed demonstrated correct knowledge.^19^ Two studies asked participants to identify the hormones “estrogen” and “progesterone” as of import to reproductive health.^20,32^ Of those two studies, only one provided statistical results, which stated that 30.4% and 24% of women were able to identify estrogen and progesterone, respectively, as important hormones of the reproductive system.^20^

Functional biology of the reproductive system relates to the general functions of female reproductivity. Across the six studies (33%) that assessed this topic, a percentage of women displayed correct knowledge of the number of eggs released at ovulation (20.8%), the frequency of ovulation (54.8%), ovum lifespan (46.3%), the biology behind pregnancy (53.6%) and childbirth (49%), and ovarian egg production across the lifespan (52%).^20–22,27,32,33^

### Knowledge of factors affecting reproductive health

Six of the studies reviewed (33%) investigated women’s knowledge of factors affecting reproductive health.^18,21,22,27,28,31^ The factors investigated and the average percentages of women’s correct knowledge across these studies are as follows: absent/irregular periods (70.2%),^18,27^ age-related decline (47.7%),^18,22,28,31^ alcohol consumption (74.5%),^27,31^ contraceptive use (2.9%),^28^ disease (93.4%),^21^ drastic diet changes (61.2%),^21^ irregular circadian rhythms (60.3%),^21^ long-lasting physical effort (37.5%),^21^ obesity (49.2%),^18,27,31^ smoking (81.4%),^18,21,27,31^ sexually transmitted infections (73.3%),^27,31^ stress (92.5%),^21,27^ and being underweight (73.2%).^27^ Age-related decline and smoking were the topics most questioned in the studies (30%), followed by obesity (23%), then absent periods, alcohol consumption, and sexually transmitted infections (15%). Women generally had good knowledge of the ill effects of stress, smoking, and the use of alcohol; however, knowledge of age-related decline varied greatly, with women from Japan and the United States having above-average knowledge and women in India and Thailand below average (see Table 6).

**Table 6.** Results of Subgroup Analysis of Women’s Knowledge of Factors That Affect Reproductive Health

Factors affecting reproductive health/menstruation							
	Akizuki (2023)^18^ Japan	Chawlowska (2020)^21^ Poland	Fowler (2023)^22^ United States	Lundsberg (2014)^27^ United States	Mahey (2018)^28^ India	Na Nakhon (2018)^31^ Thailand	Calculated mean percentage of correct knowledge
Absent/irregular period	67.8%^a^			72.7%			70.2%
Age-related decline	87.5%		62.4%		26%	15%	47.7%
Alcohol				69.3%		79.8%	74.5%
Contraceptive use					2.9%		2.9%
Disease		93.4%					93.4%
Drastic diet changes		61.2%					61.2%
Irregular circadian rhythm		60.3%					60.3%
Long-lasting physical effort		37.5%					37.5%
Obesity	23%			74%		50.6%	49.2%
Smoking	88.8%	91%		71.3%		74.7%	81.4%
Sexually transmitted infections				69.4%		77.3%	73.3%
Stress		95.6%		90%			92.8%
Underweight				73.2%			73.2%
Mean overall value of correct knowledge							62.8%

^a^ Percentages indicate participants with correct knowledge.

## Discussion

Reproductive health continues to be discussed as a global health priority, yet research elucidates poor knowledge in female populations.^36^ The general conclusions of this literature review relay the lackluster level of comprehension in women using descriptives such as “low,” “inadequate,” “incomplete,” “insufficient,” “limited,” “incorrect,” and “had significant gaps” in knowledge. Not a single study captured in this review concluded women had appropriate levels of reproductive and menstrual health knowledge. These conclusions were mirrored in a literature review of health literacy and women’s reproductive health, which reported that anywhere between 9% and 78% of women had less than adequate health literacy skills.^16^ Similarly, a review of adolescent women’s menstrual health literacy demonstrated a general lack of knowledge about menstruation in low-, medium-, and high-income countries, highlighting that issues related to menstrual health literacy transcend geographic location, and cultural and social status.^13^ Elevating women’s reproductive health and menstrual literacy on a global scale requires considerable efforts, as empowering women and promoting self-agency in health care are crucial for improving their dignity and quality of life.^37^

The paucity of awareness in women can be explained by multiple factors. The first being cultural and religious contexts in which menstruation and sexual health are taboo and shameful topics.^38^ A profound lack of menstrual health knowledge was observed in a population of young Saudi Arabian women, who described their scarcity of awareness as a direct cause of emotional distress, with the advent of menarche associated with bad memories and negative emotions.^39^ Feelings of shame and secrecy concerning menstruation are commonly expressed in women around the world, but especially in populations of Indigenous women, with internalized taboos, cultural confinement of “women’s business,” shared accommodation, and disposal of menstrual products contributing to the poor provision of appropriate menstrual health knowledge and hygiene.^40^ For example, Australian Indigenous women face disproportionate health outcomes, and while health promotion aspires to empower and encourage participation of these women, the subjugation of Indigenous knowledge systems and language remains a consideration when researching collaborative solutions.^41^ Comparative studies over a decade of young migrants from African and Middle Eastern countries living in Melbourne, Australia, concluded that cultural barriers and lack of education contributed to low sexual and reproductive health knowledge. These studies found that knowledge levels had not increased over time, identifying silence as the main barrier to sexual health literacy.^42^ Women of these cultural communities rarely receive information from their parents or teachers, and prefer to engage in self-directed learning via the internet for all their sexual and reproductive health information.^39^ Control over women’s access to knowledge is a recurrent theme in sexual and reproductive health literature, and these cultural and gender-based norms drive the menstrual stigma women endure.^42^ Stigma and discrimination are public health concerns and must be remedied in accordance with the human rights acts of appropriate access to health care, and to live without discrimination, though understanding localized community needs is required to inform the specific range of actions needed to address menstrual discrimination.^43^ Since menstrual health and menstrual inequity are frequently determined by social power structures, understanding menstrual health literacy requires attention to the intersectional nature of women’s health.^44^ Factors such as race, ethnicity, socioeconomic status, and gender significantly shape health outcomes. Studies show that racial-ethnic health inequalities are most pronounced among women, and assumptions that all women share the same experiences—regardless of age, culture, income, geography, or identity—ignore the diversity of their needs.^45^ This essentializing approach prioritizes gender over other crucial health determinants and marginalizes the experiences of vulnerable groups, including women from racial and ethnic minorities, low-income backgrounds, Aboriginal communities, LGBTQ+ groups, and those living with disabilities. As a result, their menstrual health needs remain underrepresented in both research and health promotion and policy.^46^

Level of education, and its translational impact on reproductive health literacy, is an important factor to consider, but especially relevant in regions in which poverty and low literacy are ongoing social issues.^47^ This review captured studies with a broad representation of levels of education. Female university students were frequently captured in this review^18,19,21,32–34^; however, the only consequential example of education improving reproductive health knowledge was seen in one study comparing students of health science disciplines with students of non-health-related disciplines.^34^ Often, higher levels of education are mistakenly assumed to equate to higher health literacy by health care providers; however, level of education is not a definitive factor in level of health literacy.^1^ Women self-educate using multiple and varied resources including the internet, social media, libraries, and community members, but especially from trusted health professionals.^48^ Health education promotion frames health care as an important resource for everyday life, and health care providers must consider the individual biopsychosocial factors of each person.^37^ However, there is an apparent lack of knowledge of what services exist and what services offer, while sensitive issues such as reproductive health are rarely self-disclosed.^49^ The benefits of health education promotion are exemplified in a follow-up study that evaluated the effectiveness of a web-based resource to improve menstrual health literacy and self-management of menstrual symptoms in young women. Sixty percent of participants expressed beneficial changes in the way they managed menstrual symptoms, half of the participants visited their general practitioner (GP) due to their involvement in the study, and the general conclusions were that menstrual health literacy was improved.^15^ Health promotion interventions such as these offer valuable insight into practical, interactive, self-led, accessible solutions to the glaring inadequacy of current efforts to improve menstrual and reproductive knowledge in populations around the world. Further research and development of educational interventions are warranted.

The experience of menarche and menstruation is often rendered as isolated events; however, menstruation is a significant, repetitive event in most women’s lifespans, and while the literature frequently documents this normal physiological process with negative perceptions, there continue to exist major gaps in the approach to research and health care management.^50^ Ovulation awareness and conception have been consistent foci of research in this field.^51^ That being so, our review found that only half of women could identify fertile signs during the ovulatory window, and less than half of women could correctly identify the timing of ovulation during their menstrual cycle. A similar result was reflected in an Australian study of women’s awareness of factors that influence fertility, with only a third of participants correctly identifying the most likely time to conceive during their menstrual cycle.^52^ Pregnancy and motherhood are often viewed as an ideal female experience across many cultures and have been well researched medically; however, this is in contrast to its reproductive counterparts (menstruation and menopause).^50^ Some research has indicated that, due to increased health promotion, health literacy potentially increases during pregnancy.^48^ This literature review supports previous discourses that argue female reproductive biology is largely underrepresented in research when compared with pregnancy and lactation, and that the lens must be focused on repairing the lag that exists in conceptual paradigms, language, and research of women’s reproductive health across the lifespan.^53^

Discourse surrounding definitions of “normal” are warranted, considering research indicates up to 30% of women will experience alterations in the volume or regularity of menstrual blood flow, and, in addition, many women will present with concomitant physical or mental symptoms (pain, dysmenorrhea, fatigue, anxiety, depression) associated with their menstrual cycle, which require attention for medical assessment.^5^ The incongruity behind women’s knowledge of dysfunctional menstruation may be explained by the findings in one study of reproductive health literacy in adolescent Australian women. The study presents astonishing information that women assemble an idea of what a “normal” menstrual cycle looks like from their own experience.^54^ In another study, 51% of Australian women presented with a menstrual dysfunction that they assumed was a normal presentation of their menstruation.^55^ As evidenced in this review, the lack of standardized outcome measurement tools used in the collection of data from women means it is difficult to assess and compare differing populations for meaningful universal data on reproductive and menstrual health literacy. The single instrument shared by just two studies in this review was the KFB scale; however, the way in which these studies presented results data meant that direct comparison between populations was prohibitive.^20,32^ This is not a unique research dilemma in women’s health research, as suggested in a systematic review of patient-based outcome measures used to evaluate cases of abnormal uterine bleeding. The review found that, of the 50 different instruments used, the majority had no documentation of reliability, precision, or feasibility.^56^ Standardizing data collection in research and clinical care is imperative to promote consistency and optimize comparative effectiveness in research.^5^

Two critical areas for menstrual health promotion in adult women remain under-addressed: (1) improving health care provider training in women’s menstrual health, and (2) increasing the efforts of health promotion and education within clinical care. Studies across European health care systems reveal that providers often lack adequate knowledge and time to manage complex menstrual health presentations.^57^ A recent investigation into provider support for patients with premenstrual dysphoric disorder (PMDD) highlighted variability in provider competencies and recommended strengthening graduate and medical curricula to better equip practitioners in PMDD evaluation and treatment.^58^ Women’s dissatisfaction with endometriosis care is also well-documented, with key concerns including unmet information needs, lack of empathetic communication, and inadequate technical proficiency among providers.^57^ Digital menstrual tracking tools have gained popularity, with users describing the benefits of record keeping, symptom management, increased self-awareness, and enhanced communication with health care professionals. One study noted that menstrual data served as “objective” evidence to support diagnosis, although apps were seen as “helpful but need to be more suitable,” and it could be argued the burden of responsibility remains with the patient and not the provider to improve health literacy.^59^ Complementary medicine modalities emphasize person-centered models of care, with naturopathy particularly guided by the principle of “physician as teacher.”^60^ Research on acupuncture for dysmenorrhea found that empowering patient–provider relationships—rooted in education and support rather than instruction—enabled women to shift from passive recipients to active participants in their care. Participants reported gaining a better understanding of their menstrual cycles and appreciated the contrast with prior experiences in conventional medical settings.^61^ Care providers, particularly practicing person-centered models of care, are central to advancing women’s menstrual health literacy, as clinical settings remain the primary context in which women seek information, support, and understanding of their health.

### Limitations

This study focused on literature from the last decade, limiting the search to primary resources across six databases, excluding gray literature and governmental resources that could have provided secondary data offering insights into global research methods on this topic. The study also does not capture the experiences of menstruating people who do not identify as female, woman, or women. A major limitation is the general lack of research on menstrual health, which hinders accurate representation of women’s knowledge and needs globally, with fewer than 4000 publications on menstruation published per decade between 1991 and 2019.^5^

## Conclusion

This review confirms prior evidence that women’s menstrual and reproductive health knowledge remains universally low. Raising menstrual health literacy is essential for improving dignity and quality of life. Cultural and social stigma continue to hinder empowerment and health care access. Prioritizing accessible education in health promotion can enhance literacy and reduce discrimination. Standardizing and modernizing women’s reproductive health care in research and practice is a crucial step toward advancing global public health and equity.

## Abbreviations Used

CKFS: Cardiff fertility knowledge scale
GP: General practitioner
HMB: Heavy menstrual bleeding
JBI: Johanna Briggs Institute
KFB: Knowledge of female body
PCOS: Polycystic ovarian syndrome
PMDD: Premenstrual dysphoric disorder
PRIMSA-P: Preferred reporting items for systematic reviews and meta-analysis protocols
RH: Reproductive health
RHKI: Reproductive Health Knowledge Index
SD: Standard deviation
STI: Sexually transmitted infection
U.S.: United States
WHO: World Health Organization
