# Supplementary material for: Empowering Women Through Knowledge: A Systematic Review of Literature on Menstrual and Reproductive Health Literacy
Source: Health Equity. 2025 Aug 14;9(1):357–74. doi: 10.1177/24731242251363080 (PMC12411900; doi:10.1177/24731242251363080)
Supplement: Supplementary Table S1 [file 24731242251363080_supplementary_table_s1.docx]

**Supplemental Information Table 1. Database search strings**

| Database | Search string |
| --- | --- |
| PubMed | ((((knowledge) OR (literacy)) OR (health literacy) AND (y_10[Filter])) AND (((menstruation[MeSH]) OR (menstrual cycle)) OR (menses) AND (y_10[Filter]))) AND (((reproductive[MeSH Terms]) OR (Fertility[MeSH])) OR (Fertility awareness) AND (y_10[Filter])) |
| Scopus  Filters:  Article  Last 10 years  English | (TITLE-ABS-KEY(knowledge) OR TITLE-ABS-KEY(literacy) OR TITLE-ABS-KEY(health AND literacy) AND TITLE-ABS-KEY(menstruation) OR TITLE-ABS-KEY(menstrual AND cycle) OR TITLE-ABS-KEY(menses) AND TITLE-ABS-KEY(reproductive) OR TITLE-ABS-KEY(fertility) OR TITLE-ABS-KEY(fertility AND awareness)) AND PUBYEAR > 2012 AND PUBYEAR < 2024 |
| ProQuest  Filters:  Scholarly Journals  Last 10 Years  Article  English  Peer reviewed  Full text  Subject: menstruation | title(knowledge) OR title(literacy) OR title(health literacy) AND title(menstruation) OR title(menstrual cycle) OR title(menses) AND title(reproductive) OR title(fertility) OR title(fertility awareness) |
| CINAHL  Filters:  Full text  Last 10 years  English  Scholarly Peer Reviews Journals | (knowledge or literacy or health literacy) AND (menstruation or menstrual cycle or menses) AND (reproductive or fertility or fertility awareness) |
| MEDLINE  Filters:  Full text  Last 10 years  English  Scholarly Peer Reviews Journals | (knowledge or literacy or health literacy) AND (menstruation or menstrual cycle or menses) AND (reproductive or fertility or fertility awareness) |
| AMED  Filters:  Full text  Last 10 years  English  Scholarly Peer Reviews Journals | (knowledge or literacy or health literacy) AND (menstruation or menstrual cycle or menses) AND (reproductive or fertility or fertility awareness) |
