# Supplementary material for: Empowering Women Through Knowledge: A Systematic Review of Literature on Menstrual and Reproductive Health Literacy
Source: Health Equity. 2025 Aug 14;9(1):357–74. doi: 10.1177/24731242251363080 (PMC12411900; doi:10.1177/24731242251363080)
Supplement: Supplementary Table S2 [file 24731242251363080_supplementary_table_s2.docx]

| Table 2. Johanna Briggs Institute Critical Appraisal for Cross Sectional Studies | | | | | | | | | |  |
| --- | --- | --- | --- | --- | --- | --- | --- | --- | --- | --- |
| Author/Date | **1** | **2** | **3** | **4** | **5** | **6** | **7** | **8** | **Total** | **Quality** |
| Akizuki, Y, 2023 | Y | Y | Y | N/A | N | N | Y | Y | 5/7 | High |
| Ameade, E, 2016 | N | Y | Y | N/A | UC | N | Y | Y | 4/7 | Medium |
| Ayoola, A, 2016 | Y | Y | Y | N/A | Y | N | Y | Y | 6/7 | High |
| Chawlowska, E, 2020 | Y | Y | Y | N/A | N | N | Y | Y | 5/7 | High |
| Fowler, C, 2023 | Y | Y | Y | N/A | Y | Y | Y | Y | 7/7 | High |
| Getahun, MB, 2020 | N | Y | Y | N/A | N | N | Y | Y | 4/7 | Medium |
| Halleran, M 2022 | Y | Y | Y | N/A | N | N | Y | Y | 5/7 | High |
| Hamdanieh, M, 2021 | Y | Y | Y | N/A | N | N | Y | Y | 5/7 | High |
| Jean Simon, D, 2023 | UC | Y | Y | N/A | UC | Y | Y | Y | 5/7 | High |
| Lundsberg, LS, 2014 | N | Y | Y | N/A | UC | N | Y | Y | 4/7 | Medium |
| Mahey, R, 2018 | Y | UC | Y | N/A | N | N | Y | Y | 4/7 | Medium |
| Marsh, E, 2014 | UC | UC | Y | N/A | N | N | Y | Y | 3/7 | Poor |
| Mengistie, D, 2023 | UC | Y | Y | N/A | UC | Y | Y | Y | 5/7 | High |
| Na Nakhon, S, 2018 | Y | Y | Y | N/A | N | N | Y | Y | 5/7 | High |
| Patra, S, 2021 | Y | Y | UC | N/A | N | N | Y | Y | 4/7 | Medium |
| Sons, A, 2023 | Y | Y | Y | N/A | N | N | Y | Y | 5/7 | High |
| Sreepoorna, P, 2020 | Y | Y | Y | N/A | N | N | Y | Y | 5/7 | High |
| Szues, M, 2017 | N | Y | Y | N/A | N | N | Y | Y | 4/7 | Medium |

Key: Y =Yes; N = No; UC = Unclear; N/A = Not applicable
